# Impact of Different Levels of iPTH on All-Cause Mortality in Dialysis Patients with Secondary Hyperparathyroidism after Parathyroidectomy

**DOI:** 10.1155/2017/6934706

**Published:** 2017-06-05

**Authors:** Qiu Ping Xi, Xi Sheng Xie, Ling Zhang, Rui Zhang, Yue Fei Xiao, Cheng Gang Jin, Yan Bo Li, Lin Wang, Xiao Xuan Zhang, Shu Tong Du

**Affiliations:** ^1^Department of Nephrology, China-Japan Friendship Hospital, Beijing, China; ^2^Department of Nephrology, Nanchong Central Hospital, Second Clinical Medical Institution of North Sichuan Medical College, Nanchong, China; ^3^Department of Nephrology, Aerospace Center Hospital, Beijing, China; ^4^School of Social Development and Public Policy, Beijing Normal University, Beijing, China; ^5^School of Management Beijing University of Chinese Medicine, Beijing, China; ^6^Department of Nephrology, Dalian University Affiliated Xinhua Hospital, Dalian, China; ^7^Department of Nephrology, The Fourth Hospital of Jilin University, Changchun, China; ^8^Department of Nephrology, Cangzhou People's Hospital, Cangzhou, China

## Abstract

**Background:**

Secondary hyperparathyroidism (SHPT) usually required parathyroidectomy (PTX) when drugs treatment is invalid. Analysis was done on the impact of different intact parathyroid hormone (iPTH) after the PTX on all-cause mortality.

**Methods:**

An open, retrospective, multicenter cohort design was conducted. The sample included 525 dialysis patients with SHPT who had undergone PTX.

**Results:**

404 patients conformed to the standard, with 36 (8.91%) deaths during the 11 years of follow-up. One week postoperatively, different levels of serum iPTH were divided into four groups: A: ≤20 pg/mL; B: 21–150 pg/mL; C: 151–600 pg/mL; and D: >600 pg/mL. All-cause mortality in groups with different iPTH levels appeared as follows: A (8.29%), B (3.54%), C (10.91%), and D (29.03%). The all-cause mortality of B was the lowest, with D the highest. We used group A as reference (hazard ratio (HR) = 1) compared with the other groups, and HRs on groups B, C, and D appeared as 0.57, 1.43, and 3.45, respectively.

**Conclusion:**

The all-cause mortality was associated with different levels of iPTH after the PTX. We found that iPTH > 600 pg/mL appeared as a factor which increased the risk of all-cause mortality. When iPTH levels were positively and effectively reducing, the risk of all-cause mortality also decreased. The most appropriate level of postoperative iPTH seemed to be 21–150 pg/mL.

## 1. Introduction

SHPT is a common and serious problem in patients with chronic kidney disease (CKD). With the increasing number of patients receiving long-term maintenance dialysis, SHPT appears more in patients receiving dialysis and eventually develops to refractory secondary hyperparathyroidism (rSHPT). Elevated serum concentrations of PTH may contribute to active vitamin D treatment resistance, bone and joint pain, pruritus, fractures, skeletal malformations, and cardiovascular calcification and are independently associated with all-cause and cardiovascular-related mortality [1–4]. It is believed that optimal levels of serum iPTH are different in various stages of CKD and demonstrated that either very low or high levels of serum iPTH are associated with the related mortality [5–7]. However, researchers are always interested in the following questions: what kind of PTX should rSHPT patients undergo and what is the best range of serum level of iPTH after the PTX. Since there has been a lack of relevant evidence, the present study was to investigate the associations between different levels of serum iPTH and the mortality of patients, after the PTX. The unique features of our study hinge on the relatively large sample size with corporation of multicenters, as well as with a long-term follow-up program.

## 2. Materials and Methods

### 2.1. Selection of Patients

Patients under study were from five hospitals, including China-Japan Friendship Hospital and the Aerospace Center Hospital in Beijing, Dalian University Affiliated Xinhua Hospital in Liaoning, the Fourth Hospital of Jilin University in Jilin, and Cangzhou People's Hospital in Hebei. Patients were followed up for 1–11 years.* Inclusive criteria on patients are as follows:* (1) patients who had received all prevalent dialysis with duration > 3 months, at any age; (2) patients with the level of serum iPTH > 800 pg/mL which met the PTX criteria and with hypercalcemia, hyperphosphatemia, or calcium × phosphorus (Ca × P) > 70 mg^2^/dl^2^ [3].* Exclusion criteria on patients are as follows:* (1) patients who did not receive dialysis but with CKD; (2) patients who had primary hyperparathyroidism; (3) patients who had received kidney transplantation; (4) patients who underwent repeated PTX; (5) patients who were lost to follow-up or with missing data.

### 2.2. Clinical Data

#### 2.2.1. Baseline Characteristics

The following data were retrieved from the patients' charts and computer-based records. Demographic details would include age, sex, primary cause of end-stage renal disease (ESRD) (diabetes, hypertension, chronic glomerulonephritis, and polycystic kidney), preoperative and postoperative PTX laboratory biochemical indexes correction of serum calcium (Ca_alb_), inorganic P, ALP, and iPTH. Number of deaths and complications which are calculated for each outcome were counted as the events, with duration of follow-up or death recorded. Patients who were followed up for more than 1 year were included in the survival analysis as truncated values.

#### 2.2.2. Laboratory Biochemical Index and Methods for Detection

All the samples were collected in the morning or before performing the hemodialysis, from the PTX database in the above said five hospitals. Laboratory indicators were referred to the criteria serum Ca 2.1–2.54 mmol/L (8.4–10.1 mg/dl). Serum Ca was adjusted for serum albumin according to an equation commonly used in the general population: adjusted Ca [Ca_alb_  (mg/dl)] = Ca  (mg/dl) + 0.8 × [(4-Serum  albumin  (g/dl)] [3], P 1.13–1.78 mmol/L (3.5–5.51 mg/dl), and ALP 40–150 IU/L, and serum iPTH (16~65 pg/mL) was detected by enzyme-linked immunosorbent assay, from The United States DLS intact-PTH 10–8000 kit.

### 2.3. Management on Postoperative Patients

PTX surgical operation was standardized in the five hospitals through a training program and a unified postoperative treatment process was implemented and managed by the China-Japan Friendship Hospital. The procedures would include total parathyroidectomy (tPTX), subtotal parathyroidectomy (sPTX), and tPTX with total autotransplantation (tPTX + AT). The Kidney Disease Outcomes Quality Initiative (K/DOQI) clinical practice guidelines were referred to as the post-PTX management processes, which also include daily monitoring of serum Ca once or twice a week after surgery. Patients were provided with foods, rich in protein, calcium, and phosphorus. Post-PTX, orally administered drugs would include calcium carbonate and calcitriol. We also adjusted the dose according to each serum calcium level until calcitriol reached 4 ug per day [3]. When the serum Ca_alb_ level was lower than 1.8 mmol/L, we started to inject calcium gluconate between 1 mg and 2 mg per kg per hour. When the serum Ca_alb_ level was greater than 1.8 mmol/L, the dose of calcium supplements was gradually reduced. Appropriately, we used a high dialysate calcium concentration between 1.75 and 2.25 mmol/L after the PTX.

### 2.4. Grouping under Different Levels of Serum iPTH

All the postoperative PTX-related data were entered into the follow-up database. All patients were divided into four groups according to the levels of serum iPTH, one week after surgery, regardless of the PTX. The groupings were A: iPTH ≤ 20 pg/mL (*n* = 205, 50.7%), B: iPTH 21–150 pg/mL (*n* = 113, 28.0%), C: iPTH 151–600 pg/mL (*n* = 55, 13.6%), and D: iPTH > 600 pg/mL (*n* = 31, 7.7%).

## 3. Statistical Analysis

Stata 12.0 program was performed for statistical analysis. Normal distribution values were expressed as mean ± standard deviation (SD), and *t*-test was used for comparison of two means of independent samples. The abnormal distribution values were expressed as median (interquartile range) (*M* (QL, QU)) and rank-sum test was used to compare the differences between groups. Qualitative data were expressed as the number of cases (rate) and Chi-square test was employed for comparison of qualitative variables. Survival analysis was performed with Cox regression while survival curves were represented with Kaplan–Meier curve. Statistically significant threshold was considered as *P* < 0.05.

## 4. Results

### 4.1. Basic Information

Between January 2004 and December 2014, 525 patients underwent the PTX. Under the exclusion criteria, 404 patients (215 males and 189 females) were qualified for analysis (median age as 47.32 ± 11.52 years, with median dialysis vintage as 100.97 ± 54.55 months). Among them, 396 (98%) patients were with hemodialysis while another 8 (2%) patients were with peritoneal dialysis. The primary cause of ESRD would include chronic glomerulonephritis (*n* = 203, 50.2%), hypertensive nephropathy (*n* = 25, 6.2%), polycystic kidney disease (*n* = 20, 5.0%), and diabetic nephropathy (DN) (*n* = 3, 0.7%), and the rest (*n* = 48, 11.9%) were CKD caused by chronic pyelonephritis, Chinese herbal medicine-related nephropathy, or patients with unexplainable causes (*n* = 105, 26.0%) (Table 1).

**Figure 1:**
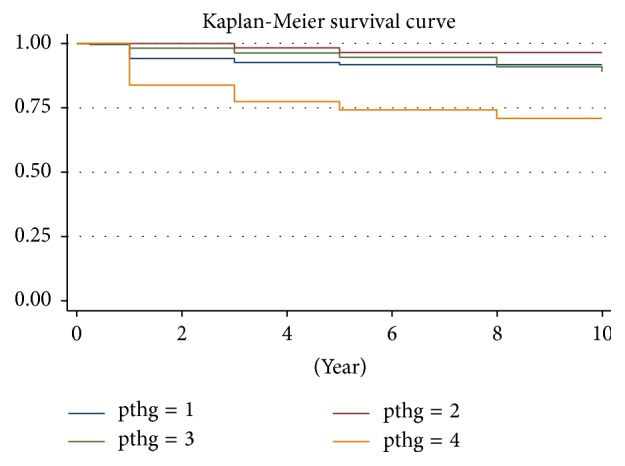
Unadjusted Kaplan–Meier survival curve. Note: pthg: 1 = iPTH ≤ 20 pg/mL; 2 = iPTH 21–150 pg/mL; 3 = iPTH 151–600 pg/mL; 4 = iPTH > 600 pg/mL.

During the 1–11-year follow-up period, the median duration was 2.3 ± 2.03 years, with an all-cause mortality rate as 8.91% (*n* = 36). In the follow-up period, 7 patients lived for over 10 years, 8 for 8–10 years, 52 for 5–8 years, 146 for 3–5 years, and 372 for 1–3 years. No case of death was observed in the first week after the PTX. We mapped the unadjusted Kaplan–Meier curve according to all-cause mortality of the patients. During the follow-up period, we found that, with patients with SHPT in different iPTH groups, the survival outcomes were different after the PTX. Among them, patients in group B (iPTH 21–150 pg/mL) had the best outcomes while group D (iPTH > 600 pg/mL) had the worst (*P* < 0.05). Results of this study showed that all-cause mortality had been significantly decreased after the PTX, which also improved the long-term survival outcomes in dialysis patients with SHPT (Figure 1).

### 4.2. Relationship between Different Groups of iPTH and the All-Cause Mortality

We used the following criteria as the basis of grouping iPTH was administered for one week after PTX to compare the mortality rates of patients in different groups. The all-cause mortality rates appeared as 8.29% (17/205) in group A (iPTH ≤ 20 pg/mL), as 3.54% (4/113) in group B (iPTH 21–150 pg/mL), as 10.91% (6/55) in group C (iPTH 151–600 pg/mL), and as 29.03% (9/31) in group D (iPTH > 600 pg/mL) (*P* < 0.05), respectively. As a result, all-cause mortality in group D appeared the highest with statistical significance (*P* < 0.05). When calculating the causes of death, we noticed that 33 were due to cardiovascular events, 2 with cancers, and 1 with some kind of infection.

We used logistic regression model to set group A as the reference to compare the risk ratios on all-cause mortality, between different groups.  Table 2 showed the multivariable adjusted odds ratio (OR) and 95% CI associated with the different groups of iPTH. Group D (OR = 5.17, 95% CI 1.93–13.88, *P* < 0.05) showed statistically significant difference with other groups but not with groups B (OR = 0.42, 95% CI 0.13–1.29, *P* > 0.05) or C (OR = 1.35, 95% CI 0.49–3.69, *P* > 0.05) (Table 2). Same results were obtained by Cox proportional hazards regression model. Data from further analysis revealed that the risk factors in group D (HR = 3.45, 95% CI 1.49–7.99, *P* < 0.05) were significantly different but neither existed in groups B (HR = 0.57, 95% CI 0.19–1.72, *P* > 0.05) or C (HR = 3.45, 95% CI 1.49–7.99, *P* > 0.05) (Table 3). The adjusted results demonstrated a significant increase in HR, associated with group D iPTH > 600 pg/mL but not with other groups. We observed an increase in the HR on deaths, with high iPTH.

For all-cause mortality, data from univariate analysis confirmed that factors as age (HR = 1.06, 95% CI 1.03–1.09, *P* < 0.05) and dialysis vintage^2^ (HR = 1.0006, 95% CI 1.000–1.001, *P* < 0.05) were of significant importance. We also found that factors as gender, primary cause of ESRD, and therapies under different sorts of dialysis did not show significant correlations with death events.

**Figure 2:**
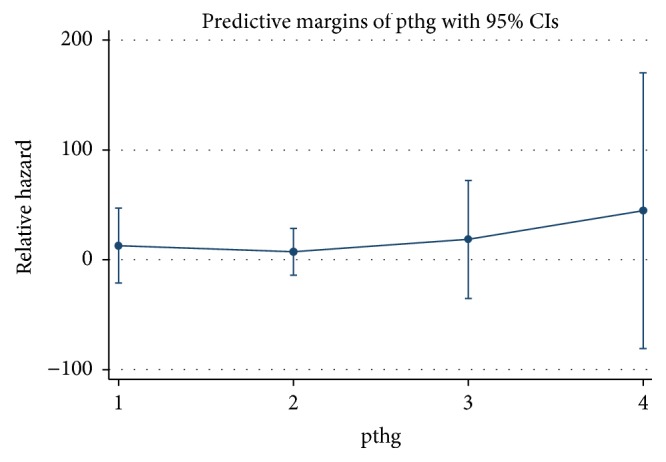
Multivariate adjusted hazard ratio comparison between different groups. Note: pthg: 1 = iPTH ≤ 20 pg/mL; 2 = iPTH 21–150 pg/mL; 3 = iPTH 151–600 pg/mL; 4 = iPTH > 600 pg/mL. Adjusted for age, gender, dialysis vintage, primary cause of end-stage renal disease, serum Ca_alb_, P, and ALP.

We used the predictive margins to analyze the relative hazard of mortality.  Figure 2 showed the comparison on the risks of all-cause mortality in different iPTH groups. We sorted the HR values from low to high as groups B, A, C, and D. Group D had the highest HR value (*P* < 0.05), with statistical significance, while the others did not. We concluded that the postoperative serum iPTH levels and the risks of death presented U-shape trends. However, only group D was with statistically significant difference.

### 4.3. Postoperative Survival in Patients under Follow-Up Program

**Figure 3:**
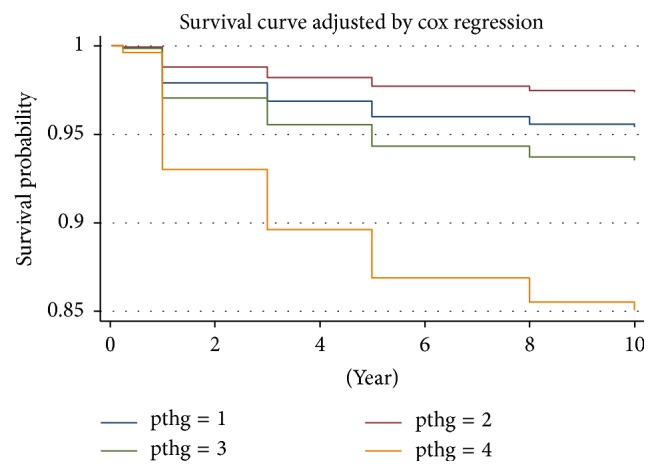
Cox proportional hazards regression model survival curves. Note: pthg = 1: A: iPTH ≤ 20 pg/mL; pthg = 2: B: iPTH 21–150 pg/mL; pthg = 3: C: iPTH 151–600 pg/mL; pthg = 4: D: iPTH > 600 pg/mL. Adjusted for age, gender, dialysis vintage, primary cause of end-stage renal disease, serum Ca_alb_, P, and ALP.

Patients were followed for different time spans after the PTX with different period as 1 week, 3 months, 1 year, 3 years, 5 years, 10 years, or more. Four groups of patients showed significant decreases on serum iPTH, after the PTX. Under the early follow-up program, four groups of patients showed different degrees of hypocalcemia postoperatively, during hospitalization. Hypocalcemia could be recovered or partially remitted by intravenous or oral calcium supplements. After the PTX, all patients showed different degrees of improvement or complete remission on bone pain, pruritus, and ectopic calcification during the follow-up period. When using the Cox proportional regression model to analyze the multivariable adjusted results, the increase of HR on death was noticed in patients of group B (iPTH 21–150 pg/mL). Patients in group D (iPTH > 600 pg/mL) showed the worst outcomes and in group C (iPTH 151–600 pg/mL) showed the poor outcomes. Prognosis of patients in group A (iPTH < 21 pg/mL) stood the second place among all the groups (Figure 3). Findings from our study suggested that, in CKD patients who kept receiving PTX hemodialysis (CKD5D) or with iPTH > 600 pg/mL, all-cause mortality on multivariables would significantly increase which were associated with the quality of life (*P* < 0.05) of the patients.

## 5. Discussion

In this study, more patients with chronic glomerulonephritis (50.2%) were seen than the ones with DN (0.7%). Chronic glomerulonephritis seemed one of the primary causes for ESRD while DN was less commonly seen in China [8]. Jiang et al. [9] retrospectively analyzed the clinical features of 496 patients with SHPT and found that chronic glomerulonephritis was the major primary cause of SHPT and DN was only 1.2%. The low prevalence of SHPT in patients with DN might be caused by the direct suppressive effect of high glucose concentration on PTH secretion from the parathyroid cells [10].

It is recognized that SHPT is a common complication of chronic renal failure. In the early stage of CKD, in order to adapt for the disorders of bone-mineral metabolism, parathyroid would excessively secrete the PTH, causing the disorders of bone-mineral metabolism to make the burden on cardiovascular systems aggravated and increased. SHPT is controlled under the treatments of phosphate binders, vitamin D analogs, or calcimimetics. With long-term uremia, patients would develop resistance to treatments and finally require PTX to participate. This can prevent the development of skeletal malformations and metastatic calcification, such as cardiovascular calcification. Known to each stage of CKD, the best serum PTH levels were considered different. Findings from our studies suggested that both extra high or low PTH could increase the mortality in CKD patients [5, 11, 12].

Because of the high variability in the number and location of parathyroid glands, they are difficult to be completely removed. Eventually, SHPT appears to be of high incidence in ESRD patients. Even after the PTX, the incidence remains relatively high [13, 14]. Chen et al. conducted a meta-analysis of randomized and prospective or retrospective studies. The results indicated that sPTX and tPTX + AT were equally successful in preventing recurrent SHPT and improving serum Ca, P, and PTH [13–16]. Sharma et al. [12] identified 150 patients under dialysis who underwent near-total parathyroidectomy (NTPTX) and were compared with 1,044 nonoperated control patients. The results indicated that NTPTX was associated with a significant reduction in the long-term risk of deaths, in patients receiving dialysis. However, in these studies, the sample size not only was small but lacked the evaluation through prospective, long-term follow-up programs on the survival quality. However, researchers always study the following questions: what kind of PTX should rSHPT patients undergo and what is the best range of serum level of iPTH after the PTX. In China, high incidence of SHPT has been seen [10]. Our research findings showed that, in 404 patients who were with PTX, the amount of preoperative iPTH reached 1950.44 ± 42 pg/mL which called for more positive PTX treatment to be carried out.

In 2008, the Dialysis Outcomes and Practice Pattern Study (DOPPS) identified the optimal PTH level as 101–300 pg/mL, with mortality risk being the lowest under this range [11], which was consistent with the 2003 K/DOQI recommended guidelines [3]. Another study presented the results that iPTH > 600 pg/mL could increase the risk of mortality [11], which was consistent with the results of our study. However, this study was mainly aimed at the patients with different stages of CKD5D, but our research of interest was focused on the patients who were receiving dialysis after the PTX therapy. Our results showed that in patients with stage of CKD5 who underwent PTX and with iPTH > 600 pg/mL the risk on all-cause mortality would increase (*P* < 0.05). In 2005, the DOPPS study suggested that when compared with PTH at 150–300 pg/mL level, the risk on all-cause mortality appeared to be higher than on the level of PTH 301–450 pg/mL. PTH > 600 pg/mL was also associated with higher risk on both cardiovascular and the all-cause mortality, even during hospitalization under cardiovascular disease-related diseases. PTH < 50 pg/mL seemed to be associated with mortality. Extra low or high PTH levels were associated with adverse outcomes [5].

Fernandez-Martin et al. examined 6797 adult patients with hemodialysis. They found that either high or low PTH was associated with high risk of mortality. Serum iPTH 398 pg/mL was associated with the minimum relative risk of mortality. Based on the published risk values on mortality, the lowest value being adopted was 168–674 pg/mL, for serum iPTH [6].

Only few studies specifically targeted on the value of PTH levels had been carried out. Considerable studies from home or abroad confirmed that SHPT patients could improve the survival outcomes after the PTX. Results from studies also showed that when patients underwent PTX, the levels of serum phosphorus, serum calcium, Ca × P, and iPTH were significantly reduced, ending in an effective improvement of quality of life on patients [12, 17, 18].

Komaba et al. [7] analyzed the data on 114,064 patients under maintenance of hemodialysis and evaluated the associations between the severity of SHPT and history of PTX, on both 1-year all-cause and cardiovascular mortality rates. They found that PTH < 60 pg/mL and PTH > 500 pg/mL could significantly increase both the all-cause and cardiovascular mortality rates. The results also showed that when compared to patients with iPTH > 300 pg/mL, those with the level of iPTH < 60 pg/mL might have a significant survival advantage. In our study, we noticed that low iPTH level (iPTH ≤ 20 pg/mL) had a tendency of increasing the risk of mortality while the level of iPTH in 21–150 pg/mL could reduce the risk for both all-cause and cardiovascular mortality rates in patients under PTX dialysis.

Since the sample size was not large enough in our long-term follow-up study, no significant statistical differences were noticed. Rhee et al. [19] claimed the persistent low iPTH (iPTH ≤ 60 pg/mL), an independent risk factor for both aortic arch calcification and mortality in hemodialysis patients. Jean et al. [20] suggested that the very low appearance of PTH level (around 10%) was associated with a significantly higher risk of mortality among non-PTX hemodialysis patients. PTH < 50 pg/mL was considered to be associated with adynamic bone disease and both of them were related to an increased risk on developing cardiovascular calcification and mortality in hemodialysis patients. Attention needs to be paid not only to high PTH but also to low PTH since both of them might influence the quality of life of patients.

## 6. Conclusion

Results from our study showed that, during the long-term follow-up period, all-cause mortality was associated with different iPTH levels in dialysis patients with SHPT, followed by the PTX. After the PTX, iPTH > 600 pg/mL seemed to be associated with the risk of all-cause mortality. When iPTH levels were positively and effectively decreasing, the risk of all-cause mortality also reduced. The most appropriate level of iPTH was found as 21–150 pg/mL after the PTX. We noticed that both the iPTH levels and the risk of mortality were under U-shapes. Unfortunately, because of the small sample size and inadequate number of patients that completed the whole course of the study, our findings were not able to reach the statistical significance as expected. Sampling error seemed another shortcoming of this study. However, we are planning to continue the study through increasing the sample size to confirm the postoperative best iPTH levels under the PTX and associations with the quality of life.

## Figures and Tables

**Table 1:** Patients characteristics by baseline iPTH category (*n* = 404).

Characteristics	iPTH ≤ 20 pg/mL	iPTH 20–150 pg/mL	iPTH 151–600 pg/mL	iPTH > 600 pg/mL	Total
Gender (male)	112 (54.6%)	58 (51.2%)	24 (43.6%)	21 (67.7%)	215 (53.2%)
Age (year)	47.36 ± 11.7	46.58 ± 11.46	48.87 ± 9.66	46.9 ± 13.79	47.32 ± 11.52
Dialysis vintage (month)	106.59 ± 58.59	96.98 ± 50.85	90.58 ± 50.40	96.81 ± 44.76	100.97 ± 54.55
Primary cause of ESRD					
Chronic glomerulonephritis	95 (46.3%)	61 (54%)	27 (49.1%)	20 (64.5%)	203 (50.2%)
Diabetic nephropathy	1 (0.5%)	1 (0.9%)	0 (0%)	1 (3.2%)	3 (0.7%)
Hypertensive nephropathy	21 (10.2%)	2 (1.8%)	1 (1.8%)	1 (3.2%)	25 (6.2%)
Polycystic kidney	13 (6.3%)	4 (3.5%)	1 (1.8%)	2 (6.5%)	20 (5%)
Other	25 (12.2%)	14 (12.4%)	6 (10.9%)	3 (9.7%)	48 (11.9%)
Unknown	50 (24.5%)	31 (27.4%)	20 (36.4%)	4 (12.9%)	105 (26%)
Serum Ca_alb_ (mmol/L)	2.56 ± 0.24	2.54 ± 0.22	2.67 ± 0.77	2.52 ± 0.16	2.29 ± 0.37
Serum P (mmol/L)	2.19 ± 0.58	2.21 ± 0.48	2.17 ± 0.48	2.38 ± 0.40	1.58 ± 0.63
Serum ALP (IU/L)	589.53 ± 543.19	604.79 ± 662.54	525.34 ± 471.26	454.17 ± 402	578.88 ± 670.32
Serum iPTH (pg/mL)	1926.76 ± 933.15	1938.70 ± 803.63	1967.75 ± 717.85	2119.09 ± 716.01	1950.44 ± 854.42
Death (*n*, %)	17 (8.29%)	4 (3.54%)	6 (10.91%)	9 (29.3%)	36 (8.91%)

Mean ± standard deviation is described if the variable is normally distributed.

**Table 2:** Results of baseline multivariate logistic regression model for all-cause mortality.

Parameter	OR	Standard error	*P* value	95% confidence interval
iPTH				
Group B 21–150 pg/mL	0.42	0.24	0.13	0.13~1.29
Group C 151–600 pg/mL	1.35	0.69	0.56	0.49~3.69
Group D >600 pg/mL	5.17	2.6	0.001	1.93~13.88
Age	1.07	0.02	0.000	1.04~1.11

Adjusted for age, gender, dialysis vintage, primary cause of end-stage renal disease, serum Ca_alb_, P, and ALP.

**Table 3:** Results of baseline multivariate Cox regression and competing risk regression analysis for all-cause mortality.

Parameter	HR	*P* value	95% confidence interval
iPTH			
Group B 21–150 pg/mL	0.57	0.32	0.19~1.72
Group C 151–600 pg/mL	1.43	0.46	0.55~3.68
Group D >600 pg/mL	3.45	0.004	1.49~7.99
Age	1.06	0	1.03~1.09
Dialysis vintage^2^	1.0006	0.04	1.00~1.001

Adjusted for age, gender, dialysis vintage, primary cause of end-stage renal disease, serum Ca_alb_, P, and ALP.
